# Factors Explaining Nurses’ Satisfaction with the Performance of Their Duties in Canada

**DOI:** 10.1177/08445621261418069

**Published:** 2026-02-09

**Authors:** Paul Jacob, Rein Lepnurm, Ata ur Rehman Quraishi, Joanne Whitty-Rogers, Roy Dobson

**Affiliations:** 1 7235 University of Saskatchewan, Saskatoon, Canada; 2 School of Public Health, 7235 University of Saskatchewan, Saskatoon, Canada; 3 Faculty of Medicine, Dalhousie University, Halifax, Canada; 4 Division of Cardiology, QEII - Halifax Infirmary, 432234 Nova Scotia Health Authority, Halifax, Canada; 5 Rankin School of Nursing, 1270 St. Francis Xavier University, Antigonish, Canada; 6 College of Pharmacy and Nutrition, 7235 University of Saskatchewan, Saskatoon, Canada

**Keywords:** Canadian nursing, organizational behavior, leadership, stress and coping, management

## Abstract

Shortages of registered nurses are frequently experienced in the provincial health care systems of Canada. The work environment needs attention to improve Nurses’ Satisfaction with the Performance of their Duties (NSPD). A hypothesized model was developed to find the factors associated with NSPD. A cross-sectional study covering two similar-sized health regions in Canada was used to test the model developed. A self-reported survey was conducted for a sample of nurses (*n* = 230) consisting of Registered Nurses (*n* = 196) and Licensed Practical Nurses (*n* = 34) working in the cardiology or stroke units in the health regions of Halifax and Saskatoon. A regression model was built to test the hypothesized model. The final model specified significant predictors of NSPD with Years in Practice as a control variable. Significant predictors explaining the variance were: Hassles (21.0%, *β* = −0.098, *p* = 0.022); Positive Attitude (21.2%, *β* = 0.108, *p* = 0.011); Unit Organization (14.4%, *β* = 0.162, *p* = 0.003); Leader Actions (3.1%, *β* = 0.133, *p* = 0.007); Objective Culture (1.5%, *β* = 0.167, *p* = 0.003); Fulfillment (7.2%, *β* = 0.251, *p* < 0.000); and Recognition (4.2%, *β* = 0.295, *p* < 0.000). The final model explained 72.6% of the variation in SPD. The evidence from this study provides insight into factors associated with NSPD. Application of leadership, motivation, and organizational culture theories to manage the work environment of hospitals to improve NSPD has the potential to alleviate current and projected nursing shortages, thus securing an experienced and satisfied nursing workforce in hospitals.

## Background

Nurses represent nearly half of the health care workforce in Canada, and the largest group of providers of patient care in large health care organizations (Canadian Institute for Health Information, 2024). The increasing need for health care services by an aging population is placing high demands on all health care professionals, especially nurses (Statistics Canada, 2017). Over the past decade, nursing shortages have become chronic in both industrialized and developing countries (Lu et al., 2005; Nei et al., 2015; Rosseter, 2014) and retention of nurses has become a global issue (Kingma, 2001; Marć et al., 2019; World Health Organization, 2006a, 2006b). Among nurses, the tendency to leave is primarily related to pay, opportunities for promotion, workload, and stress (Flinkman & Salanterä, 2015; Zeytinoglu et al., 2007), and secondarily related to satisfaction with their supervisor and the health care organization (El-Jardali et al., 2009). In addition, insufficient education and inadequate support for new nursing graduates is stressful for these individuals and generates major risks to patient safety, and can cause many new graduates to leave the nursing workforce prematurely (Canadian Association of Schools of Nursing, 2021).

Nursing is considered a stressful occupation as nurses often provide care to patients suffering from severe illnesses, some of them dying, on a daily basis (B. Hayes et al., 2015; Shen et al., 2005; Sveinsdottir et al., 2006). In addition to human suffering, nurses must deal with long working hours, shift rotations over 24 hours, including weekends and holidays, physical demands, inadequate staffing, and challenging interpersonal interactions (Gelsema et al., 2006; McGrath et al., 2003; Nowrouzi et al., 2015). As a reslt, nurses can experience significant stress-related issues such as: burnout, greater use of sick days, and higher turnover rates compared to the general work force (Clegg, 2001; Kirkcaldy & Martin, 2000). Stress at work can also cause hostility toward co-workers, and ultimately cause reductions in both productivity and the quality of care provided to patients (Mosadeghrad et al., 2011). Other researchers report that nurses who can separate work from their homelife, limit their connection with patients, and find ways to find closure from death and negative outcomes are in a better position to cope with emotional distress and hardship (Badu et al., 2020). They further found that using mindful practices helped nurses lower workplace pressure.

The pressures generally experienced by those working within health care systems also affect job satisfaction (Al-Hamdan et al., 2017; Regan et al., 2016; Shamian et al., 2002). Job satisfaction among nurses is important to all health care stakeholders in that the job satisfaction of nurses also affects the quality and safety of patient care (Boamah et al., 2017). Most studies on job satisfaction among nurses identify a variety of contributing factors including: working environment, variety of work, workload, job requirements, organizational policies, supervisor and mentor support, interpersonal communication and collaboration, professional practice and autonomy, opportunities for advancement, respect or status, recognition, work-life balance, pay, and fairness (Knoop, 1994; Sengin, 2003).

Two seminal meta-analyses by Blegen in 1993, and by Irvine and Evans in 1995, laid the foundation for assessing satisfaction with nursing careers, finding that stress, organizational commitment, and relations with leaders were the strongest predictors of job satisfaction (Blegen, 1993; Irvine & Evans, 1995). Subsequently, researchers found that job satisfaction among nurses working in acute care hospitals was related to: the organizational environment and working conditions, perceptions of the job, role conflict, ambiguity, commitment to the profession, stress on the job, supervision and communications with employes, recognition and expressions of appreciation of its employes, and opportunities for personal growth and promotion, along with pay, fringe benefits, and security (Lu et al., 2019).

Satisfaction with the performance of their duties is an integral component when assessing overall job satisfaction among nurses, and increasingly seen to improve retention and limit job turnover rates (Cho & Kim, 2022). The job satisfaction literature suggests distress, organizational culture, leadership, unit and organizational support, professional equity, the physical environment, and co-workers are key factors contributing to a worker's satisfaction with the performance of their duties; however, the concept has not been explored independently. This paper will examine the contribution of a range of factors associated with nurses’ satisfaction with the performance of their duties as reported by nurses in two medium-sized cities in Canada.

## Methods

For this study it was hypothesized that the following factors: Daily Distress (DD), Organizational Culture (OC), Unit Support (US), Leadership (L), Professional Equity (PE), and Quality Rating (QR) are associated with Nurses’ Satisfaction with the Performance of their Duties (NSPD). Contributing factors were investigated by the authors using a 13-item scale incorporating intrinsic facets of job satisfaction seen to reflect NSPD (dependent variable). NSPD items included: adequacy of resources, cohesiveness of the work group, motivation with work, task variety, autonomy, positive affect, interactions with physicians, interactions with management, nurse-patient relationship, unit administration, role clarity, career orientation, and self-image of performance.

The NSPD scale consisted of two distinct factors from a three-factor career satisfaction scale previously developed by Lepnurm and colleagues between 2005 and 2012 (Lepnurm et al., 2006, 2007, 2012, 2015). The first factor contained six items related to the performance of administrative duties, while the second factor included seven items related to performance of clinical duties. The third factor, consisting of five items related to personal satisfaction, was not included in this study as it was seen as less aligned to the concept of satisfaction with performance. As well, the shorter NSPD scale was viewed as easier to administer, thereby increasing the probability of getting well thought out responses from nurses when compared to the longer career satisfaction scale.

The independent variables consisted of, or were modified from scales previously developed by Lepnurm and associates between 2005 and 2012. The DD scale was modified from a scale originally developed for an earlier study (Lepnurm et al., 2009), and contained four sub-scales: Exhaustion; Moral Distress; Hassles; and Positive Attitude. The US scale, also developed for a previous nursing study (Lepnurm et al., 2012), contained two sub-scales: organization of the unit (UnitOrgn) and professional development (UnitDev). The OC scale was modified from a scale developed for the same study (Lepnurm et al., 2012), and contained three sub-scales: Behavioural Culture, Objective Culture, and Use of Sick Time. The L scale was developed for this study and contained three sub-scales: Values of Leaders (LValues), Integrity of Leaders (LIntegrity), and Actions of Leaders (LAction). Similar to other scales used for this study, the PE scale was originally developed for a national study of Canadian physicians (Lepnurm et al., 2006) and subsequently modified for a second study (Lepnurm et al., 2012), and contained three equity sub-scales: Pay, Fulfillment, and Recognition. The QR scale contained two sub-scales that measured the quality of people and the quality of infrastructure using a series of percentage scales developed in studies of physicians and nurses (Lepnurm et al., 2006, 2007, 2012, 2015).

### Study Design and Data Collection

A twin-site, full census cross-sectional design was used to test the hypothesized model. This model specifically identified factors affecting nurses’ satisfaction with the performance of their duties (NSPD) among nurses in Canada by studying two geographically distinct, university-based, health care organizations operating in health regions of similar size; the Saskatoon Health Region (SHR) located in Saskatoon, Saskatchewan and the Capital District Health Authority (CDHA) located in Halifax, Nova Scotia.

All nurses in the cariology and stroke units within the two health regions were invited to participate. As part of the recruitment process, members of the research team held information sessions on each unit to promote the study and answer any questions about the study from the nursing staff. Nurses wishing to participate in the study were provided with a paper questionnaire and given time at work to complete the questionnaire. Nurses also received an envelope in which to place the completed questionnaire. To ensure anonymity, nurses placed their sealed envelopes in a drop box provided on each unit. Data collection was carried out in three waves, with each wave lasting three weeks.

### Sample Size

Sample sizes for regression modeling were based on a number of factors including desired power, alpha level, number of predictors, and expected effect sizes (Tabachnick & Fidell, 2014). The guidelines used in regression modelling included: assumption of a medium-sized relationship between independent and dependent variables, alpha value of 0.05, and Beta value of 0.20 (Green, 1991; Tabachnick & Fidell, 2014). Based on six independent variables (IVs) (i.e., predictors) to explain the dependent variable (DV) (i.e., Nurses’ Satisfaction with Performance of their Duties—NSPD), the number of cases (n) was determined by use of equation 3.1, where m is equal to the number of IVs to be included in the regression model.

(3.1) $$\begin{array}{rl} & 50+8m=n\\ & \mathrm{Therefore};\\ & 50+(8*6)=n\\ & 98=n \end{array}$$

When testing for correlations among IVs, the number of cases was determined by equation 3.2.

(3.2) $$\begin{array}{rl} & 104+m=n\\ & \mathrm{Therefore};\\ & 104+6=n\\ & 110=n \end{array}$$

### Eligibility Criteria

Study subjects consisted of the nursing staff of the cardiology and stroke units within hospitals located in the Saskatoon and Halifax health regions. All Registered Nurses (RN) and Licensed Practical Nurses (LPN)^1^ working at least 0.5 full-time equivalent, for a minimum of one year, delivering patient care on either the cardiology or stroke units were invited to participate in study. Nurses were excluded if they were on vacation, parental leave, or other leaves of absence. Nurses with significant administrative duties were also excluded.

### Data Analysis

A hypothesized model of the relationship between DD, OC, US, L, PE, QR, and NSPD was developed and tested using multiple linear regression modeling. While regression modeling allows for the measurement of the effect size of multiple independent variables, causation could not be determined due to the cross-sectional design of the study.

## Results

### Response Rates and Descriptive Statistics

In total, 367 nurses consisting of 317 RNs and 50 LPNs were eligible to participate in the study with 165 nurses from Halifax and 201 from Saskatoon. A total of 230 nurses (196 RN, 34 LPN) returned completed questionnaires for an overall response rate of 62.7%. In Halifax, 116 nurses (97 RNs and 19 LPNs) returned completed questionnaires for a response rate of 70.3%, and in Saskatoon, 114 nurses (99 RNs and 15 LPNs) completed questionnaires for a response rate of 57.7%. Questionnaires were completed by 125 nurses (59.0%) on the cardiology units and 105 (67.7%) on the stroke units.

The average age reported by nurses was 37.7 years with average years in practice just over fourteen (Table 1). The average age and years in practice were lowest in the cardiology unit of the SHR and highest in the stroke unit of the CDHA. Compared to RNs, the average age of LPNs (39.6 years) was more than two years older. There was little difference in years in practice with LPNs averaging 14.4 years and RNs averaging 14.1 years.

**Table 1.** Age and Years in Practice by Unit Type/Region and Nursing Qualification.

Unit and Region	N	Age				Years in Practice			
		Mean	S. D.	Min.	Max.	Mean	S.D.	Min.	Max.
IN CU-CCU—Halifax	33	40.4	11.7	24	65	16.7	11.5	1	43
Cardio PCU—Halifax	32	35.3	11.8	22	56	12.4	11.8	1	35
Acute Stroke PCU—Halifax	32	41.3	12.8	23	63	16.7	11.9	1	37
Rehab Stroke–Halifax	19	42.2	11.6	21	57	20.0	12.3	1	37
Cardio PCU—Saskatoon	30	32.6	8.6	23	57	8.6	7.2	1	30
CCU—Saskatoon	30	39.2	10.4	25	58	16.7	11.0	3	38
Stroke PCU—Saskatoon	54	35.9	11.3	22	60	11.7	10.2	1	35
Total	230	37.7	11.5	21	65	14.1	11.2	1	43

Nursing Qualification	N	Age				Years in Practice			
		Mean	S. D.	Min.	Max.	Mean	S.D.	Min.	Max.
Registered Nurse	196	37.4	11.4	21	65	14.1	11	1	43
Licensed Practical Nurse	34	39.6	12.2	23	63	14.4	11.7	1	37
Total	230	37.7	11.5	21	65	14.1	11.2	1	43

### Linear Regression Analysis

The initial regression model was quite robust, explaining 72.6% of variance in NSPD; however, many of the individual predictors were not significant. Backward exclusion was used to eliminate insignificant predictors. The final model consisted of significant predictors of NSPD and the control variable, years in practice (Table 2). Significant predictors included: Hassles which explained 21.0% of variance (*β* = −0.098, *p* = 0.022), Positive Attitude with 21.2% of variance (*β* = 0.108, *p* = 0.011); Unit Organization 14..4% of variance (*β* = 0.162, *p* = 0.003), Leader Actions 3.1% of variance (*β* = 0.133, *p* = 0.007), Objective Culture 1.5% of variation (*β* = 0.167, *p* = 0.003), Fulfillment 7.2% of variance (*β* = 0.251, *p* < 0.000) and Recognition 4.2% of variation (*β* = 0.295, *p* < 0.000). The parsimonious model also cumulatively explained 72.6% of variation in NSPD and is considered a large effect (Cohan, 1988).

**Table 2.** Significant Predictors of Nurses’ Satisfaction with the Performance of Their Duties (NSPD).

Model Summary (Dependent Variable: NSPD (13 Items) – All Significant Independent Variables Entered														
Model	Independent Variables	R Square	Std. Error of the Estimate	Change Statistics			Std Coeff. Beta	T	Sig.	Correlations			Collinearity Statistics	
				R Square Change	F Change	Sig. F Change				Zero-order	Partial	Part	Tolerance	VIF
1	Years of Practice	0.000	0.5678	0.000	0.032	0.858	0.067	1.851	0.062	0.003	0.121	0.067	0.968	1.031
2	Hassles	0.210	0.5040	0.210	61.663	0.000	−0.098	−2.364	0.022	−0.468	−0.161	−0.083	0.688	1.452
3	Positive Attitudes	0.432	0.4241	0.212	84.573	0.000	0.108	2.542	0.011	0.538	0.184	0.099	0.640	1.559
4	Unit Organization	0.576	0.3727	0.144	47.645	0.000	0.162	3.041	0.003	0.664	0.187	0.112	0.468	2.157
5	Leader Actions	0.607	0.3696	0.031	15.248	0.018	0.133	2.781	0.007	0.522	0.178	0.096	0.594	1.692
6	Objective Culture	0.612	0.3650	0.015	8.720	0.004	0.167	2.914	0.003	0.560	0.191	0.112	0.611	1.642
7	Fulfillment Equity	0.684	0.3273	0.072	54.067	0.000	0.251	5.347	0.000	0.660	0.334	0.201	0.554	1.813
8	Recognition Equity	0.726	0.3066	0.042	31.802	0.000	0.295	5.650	0.000	0.726	0.340	0.197	0.476	2.114

Dependent Variable: Nurses’ Satisfaction with the Performance of their Duties (13 items).

Explanatory variables were measured using either six- or seven-point scales, and as such, the unadjusted R^2^ was used. The standard error of the estimate was relatively small and decreased as more variables were added to the model. Zero-order correlations of the independent variables with the dependent variable were all high, supporting their use in the regression model. Partial correlations increased as the number of variables entered were controlled for, and the part correlations of the variables increased steadily from 0.067 to 0.197. After all eight independent variables were entered, and controlling for the variance of the first seven, the partial correlation between Recognition (eighth independent variable) and NSPD was 0.340 compared to the zero-order correlation of 0.726. The unique or part correlation of Recognition with NSPD was 0.197. Examining the part correlations of the independent variables showed that adding the control variable resulted in small incremental significant contributions. Considerable shared variance was observed among the equity dimensions of fulfillment and recognition; however, the shared proportion was less than 0.35.

Additional regression analysis was conducted by health region (SHR and CHDA) and by clinical units (MI and CVA). A more granular analysis was not initiated due to the rule of thumb ratio of a minimum of 20 cases per explanatory variable (Austin & Steyerberg, 2015). Years in Practice as the control variable and the initial twelve independent variables were entered with insignificant variables eliminated in all the analyses. Using all respondents resulted in a model with seven explanatory variables (Hassles, Positive Attitude, Unit Organization, Leader Actions, Objective Culture, Fulfillment Equity and Recognition Equity) with a cumulative R square of 0.726 (Table 3).

**Table 3.** Comparing Regressions by Region/District and by Type of Clinical Condition.

Summaries of Regression Models Comparing All Respondents with Regions and Clinical Units															
	All Respondents			SHR			CDHA			MI			CVA		
Predictors	R Square	Std Coeff Beta	Sig.	R Square	Std Coeff Beta	Sig.	R Square	Std Coeff Beta	Sig.	R Square	Std Coeff Beta	Sig.	R Square	Std Coeff Beta	Sig.
Years in Practice	0.000	0.067	0.062	0.001	0.032	0.530	0.007	0.122	0.020	0.020	0.151	0.003	0.000	−0.076	0.037
Hassles	0.210	−0.098	0.022				0.291	−0.158	0.009	0.186	−0.137	0.014	0.240	−0.108	0.012
Positive Attitudes	0.432	0.108	0.011												
Unit Organization	0.576	0.161	0.003										0.476	0.164	0.002
Unit Development							0.503	0.360	0.001						
Leader Values				0.411	0.197	0.003									
Leader Integrity															
Leader Actions	0.607	0.133	0.007										0.497	0.098	0.036
Behavior Culture															
Objective Culture	0.612	0.167	0.003	0.530	0.206	0.001				0.321	0.231	0.001	0.520	0.147	0.001
Fulfillment Equity	0.684	0.251	0.000	0.641	0.249	0.001	0.694	0.348	0.001	0.611	0.364	0.001	0.661	0.288	0.001
Pay Equity															
Recognition Equity	0.726	0.295	0.000	0.733	0.407	0.001	0.731	0.250	0.001	0.711	0.398	0.001	0.716	0.329	0.001

Dependent Variable: Nurses’ Satisfaction with the Performance of their Duties (13 items).

For the SHR, only four independent variables were significant (Leader Values, Objective Culture, Fulfillment Equity, and Recognition Equity) for a cumulative R square of 0.733. For the CDHA, Years in Practice and four independent variables were significant (Hassles, Unit Development, Fulfillment Equity and Recognition Equity) for a cumulative R square of 0.731. For the MI units, Years in Practice and four independent variables were significant (Hassles, Objective Culture, Fulfillment Equity and Recognition Equity) for a cumulative R square of 0.711. For the CVA units, Years in Practice and six independent variables were significant (Hassles, Unit Organization, Leader Actions, Objective Culture, Fulfillment Equity and Recognition Equity) for a cumulative R square of 0.716.

Descriptive analysis of all the measures was conducted, but very little difference was found in any of the measures. NSPD was found to average 4.1 on a six-point scale with no significant differences by years in practice. Nurses with more than 30 years in practice reported the highest average scores at 4.3; nurses between 10 and 19 years in practice scored 4.2; the middle group with 20 to 29 years in practice scored slightly lower on average at 4.0; and nurses with less than 10 years in practice scored from 4.0 to 4.1.

## Discussion

At the time that this research was carried out, very few studies had investigated NSPD of Canadian nurses in hospital settings. The lack of models incorporating moderators of job satisfaction in nursing was a significant shortcoming in literature reviews conducted in 2005, 2012 and more recently in 2019 (Lu et al., 2005, 2012, 2019), preventing the development of interventions to improve nurse retention. The current global shortage of nurses highlights the need for understanding the impact of the factors identified as supporting NSPD. A greater understanding of factors that affect NSPD in Canadian hospitals is important as health managers face ongoing financial constraints while also seeking to maintain and improve the quality of patient care (Blegen & Mueller, 1987; Greenglass & Burke, 2001; Jones & Gates, 2007). As the impact of recent nursing shortages and financial challenges exacerbate dissatisfaction, distress, and intention-to-leave, managerial strategies to improve the working conditions of nurses is critical to ensure retention of sufficient numbers of nurses to support and improve quality of care and positive patient outcomes (Roche et al., 2015).

Nursing quality and patient outcomes are intimately intertwined. Job satisfaction of nurses and patient satisfaction with nursing care are related and two important indicators of nursing quality (Aiken et al., 2002, 2012; Laschinger & Fida, 2015; Tzeng & Ketefian, 2002). Nursing turnover rates are a recurring issue and continue to adversely affect patient outcomes and satisfaction (L. J. Hayes et al., 2006). The replacement of a nurse incurs large financial costs on a health care organization as the new position must be advertised, and candidates recruited, interviewed, selected, and trained. In addition, organizations lose relevant institutional knowledge and intellectual capital, and incur potential productivity losses as the result of nursing turnover (Contino, 2002; Jones & Gates, 2007).

Several studies have identified job dissatisfaction as being strongly associated with the intention to leave one's job (Aiken et al., 2002; Lindqvist et al., 2015; Shields & Ward, 2001; Tourangeau & Cranley, 2006). Prior to the pandemic, one in five nurses working in Canadian hospitals left their job each year resulting in an estimated per capita cost to institutions of $25,000–$67,100 (Jones, 2008; Jones & Gates, 2007; O’Brien-Pallas et al., 2010; Stone et al., 2007). It is important, therefore, to improve job satisfaction, particularly in light of the current, and what is anticipated to be a persistent nursing shortage.

### Effects of Daily Distress on NSPD

Regression analyses demonstrated that two of the four sub-scales of Daily Distress (DD), including hassles experienced by the study respondents (21.0%) and the degree to which respondents indicated a positive attitude (21.2%), cumulatively explained 42.2% of variance in NSPD. This is similar to previous findings of meta-analytic studies (Blegen, 1993; Lu et al., 2012; Zangaro & Soeken, 2007). A lack of job satisfaction is the most frequently cited reason for nurse turnover (Irvine & Evans, 1995; Yin & Yang, 2002), and there is a positive and significant relationship between job stress and intention to leave (Wu et al., 2007). There are various studies that examined nurses’ intention to quit that have demonstrated associations between lack of support and deterioration of in their emotional and mental well-being (Cavanagh & Coffin, 1992; Flinkman et al., 2010; Kalliath & Morris, 2002; Leiter & Maslach, 2009; Meeusen et al., 2011; Piko, 2006; Tyler & Cushway, 1998).

### Effect of Unit Support and Organizational Culture on NSPD

Regression modeling revealed that unit organization, a dimension of unit support, explained 14.4% of the variance (Beta = 0.162, *p* = 0.003), and the findings build upon conclusions of other studies (AlAzzam et al., 2017; Morrison et al., 1997; Mueller & Mccloskey, 1990; Spence Laschinger et al., 2016; Tovey & Adams, 1999). In this study, the unit organization subscale measured how a unit is organized to successfully complete assigned work effectively, availability of resources to adopt best practices, the ability of staff to achieve group consensus when dealing with major issues, and whether the organizational structure of the unit encourages nurses to contribute their ideas.

The organizational culture of the patient care unit has been shown to affect nurses’ behavior and perceptions of work environment (Adams & Bond, 2000; Thomas, 1992). Research has shown, for example, that Magnet hospitals^2^ have higher staff retention rates and satisfaction results for patients and nursing staff using a collaborative approach to care. These hospitals are seen to provide a helpful work environment that enables more positive outcomes (Haller et al., 2018). A study of nurses in thirty hospitals across Ireland found that positive work environments enhanced patient outcomes (Kirwan et al., 2013), and a study of Belgian hospitals consisting of 546 staff nurses from 42 units found significant relationships between the practice environment, burnout, job satisfaction, and quality of care (Van Bogaert et al., 2010, 2014).

### Effect of Leadership on NSPD

Leader Actions (LA), from the Leadership scale explained 3.1% of the variance (*β* = 0.133, *p* = 0.007) in NSPD. Many scholars investigating organizations have demonstrated that the leadership style of a supervisor affects the job satisfaction of employes reporting to them (Boamah et al., 2018; Medley & Larochelle, 1995). Transformational leadership styles are positively correlated with higher job satisfaction scores reported by unit nurses (Bawafaa et al., 2015; Boamah et al., 2018; Fallatah & Laschinger, 2016).

Similarly, there have been many studies exploring leadership style, organizational commitment, and job satisfaction. Moreover, strong positive relationships between job satisfaction and organizational commitment have been reported over time by many researchers (Atmojo, 2015; Iverson & Roy, 1994; Read & Laschinger, 2015; Saleem, 2015; Top et al., 2015; Williams & Anderson, 1991).

An Australian study concluded that the leadership style of a nursing unit manager affected staff retention (Duffield et al., 2009). Poor ratings of unit level management by nurses has also been found to negatively affect nurses’ reports of the quality of care (Van Bogaert et al., 2010, 2014).

A strong unit leader can also create a positive work environment. A study of the practice environment of nurses in Ontario, found that environments that empower nurses provide more opportunities for nurses to influence quality of patient care and ultimately job satisfaction (Laschinger & Fida, 2015). A study of 13 hospitals in western Canada involving 123 nurses found that autonomy, management support and nurse-manager relations were important factors in contributing to the job satisfaction of nurses (Smith et al., 2006). Other researchers found that authentic leadership positively affected job satisfaction and performance (Fallatah & Laschinger, 2016). The current study found that values, actions, and the integrity of leaders were positively correlated with improved Unit Support and Organizational Culture.

### Effect of Professional Equity on NSPD

Professional equity was measured using three dimensions: financial, intrinsic and recognition (Dobson et al., 2005). Intrinsic rewards are the fulfilling and gratifying aspects of the job itself. Extrinsic rewards are either tangible such as pay or intangible such as respect, appreciation, and recognition for effort. When an individual perceives a balance between contributions and rewards, the resulting equity creates a sense of satisfaction. When a balance is not achieved, the resulting dissatisfaction can lead to distress (Borkowski, 2009), and an increased intention to leave.

The regression model indicated greater relative importance of fulfillment and recognition equity, and a lesser, but still significant role for pay equity in NSPD. Previous researchers found that job satisfaction and equity were related to pay (Blegen, 1993; Perry, 1993; Singh & Loncar, 2010). A meta-analysis of nurses in hospital settings also identified fulfillment and recognition as important factors associated with job satisfaction (Blegen, 1993).

### Limitations

The limitations of the current study are primarily those associated with cross-sectional studies. Due to the fact that exposure and outcome are measured simultaneously, temporal relationship between the two could not be shown that would allow for the demonstration of causation. Furthermore, in deciding on which factors affecting NSPD should be included, some concepts were not adequately captured. The questionnaire had items on team care and coping and these were only moderately correlated with NSPD and thus, were not significant predictors. Intention to leave also was not included in the questionnaire.

The generalizability of the findings may be seen as limited due to the focus of the study on only two clinical areas, cardiology and stroke. The nature of nursing work and unique patient characteristics associated with other clinical areas might be expected to mitigate the effect size seen in this study.

While the aspects of nurses’ satisfaction with the performance of their duties captured by the 13-item scale was quite extensive, it was not exhaustive. Factors such as practicing in areas in which the nurse had limited training and/or experience due to staffing constraints, or the inability to provide more timely care due to limits to scopes of practice were not captured. The potential contribution of these and other unidentified factors to NSPD is unknown.

Additionally, psychometric surveys tend to suffer from variances that can be attributed to the methods of measurement and not the variances due to the actual constructs used to measure the concepts (Chang et al., 2010; Podsakoff et al., 2003, 2012).

In developing the questionnaire, slight differences were seen in terminology between the clinical conditions and the regions. Such differences may affect categorical variables describing the nature of the nurses’ responsibilities, the specific types of quality assurance activities carried out and the profile of duties. In an effort to mitigate this issue, eight iterations of the wording used for the psychometric measures were circulated and reviewed over a year long period among the members of the research team located in Saskatoon and Halifax to create more uniform measures.

Although the response rate for a full-census all-inclusive effort was encouraging, self-selection bias might be present whereby nurses with more confidence in the quality of their work and the work environment might have been more likely to respond to survey questionnaires. In addition, the possibility exists whereby some nurses with specific concerns related to their workplace might have utilized the study as an opportunity to highlight these issues to hospital administrators.

The response rate of nurses in this study (63.61%) was deemed sufficient and representative of the population as it was similar to previous studies (Cook et al., 2009). Overall, the results of the study should be interpreted based on the limitations discussed above, and more research, specifically longitudinal studies, is needed to mitigate these limitations and to provide more conclusive evidence on the cause-effect relationship of the factors associated with NSPD.

## Implication of Findings

### Nursing Practice and Health Care Management

The findings of this study identify characteristics of a healthy work environment and provide health care managers with important information as they work to support nurses in the performance of their duties. Leadership teams could potentially implement techniques that support authentic leadership^3^, structural empowerment initiatives^4^ that address dimensions of opportunity, and transparency in planning, as well as access to information and other resources supportive of NSPD.

Better orientation and mentoring by seasoned nurses or career coaches might improve support for the patient care unit that in turn would reduce the daily distress experienced by nurses. Providing easy access to online internal and external employe assistance programs may enable nurses to make better patient care decisions; thereby reducing daily distress levels. Effective transformational leaders value their employes enough to give them power to make decisions that achieve the objectives of the organization, thus enhancing satisfaction with their jobs; and giving rise to positive feelings about the organization (Choi et al., 2016).

Unit managers who empower staff to change the assignment of duties to ensure that growth opportunities arise from the performance of daily duties may positively affect NSPD (Morrison et al., 1997; Spence Laschinger et al., 2010). Health administrators could empower nurses to be more involved in committees at various levels such as at the unit/ward level, occupational health and safety committees and institutional-level employe wellness committees. Involvement in these committees may also improve self-esteem, boost recognition equity and thereby improve professional equity. Participation in committees of various levels may help employes learn new inter-personal and leadership skills. Inclusion of nurses in quality improvement initiatives and other workplace wellness committees may aid improvement of NSPD. Involvement in purchase decisions related to their job, such as portable monitors and other common bedside equipment also may positively affect morale.

### Nursing Practice and Policy

While this study does not provide concrete and evidence-based solutions and interventions, it does provide evidence on factors associated with NSPD that might be used by policy makers to enable hospital administrators to improve work environments. For example, allocating adequate resources to factors seen as contributing to NSPD to ensure better retention, job satisfaction, and improved quality of patient care. To improve staff morale initiatives, it is important that hospital leadership solicits recommendations from nurses themselves to improve delivery of care. Furthermore, factors identified in this study that support NSPD could be used to develop performance metrics for policy makers to assess changes in nursing staff morale and patient care quality.

### Education

Similar to other established professions, nursing students are introduced to professional work environments throughout their studies. It is important, therefore, to ensure nursing students have the knowledge and competencies needed to work in the health care setting.

Several baccalaureate degree pathways have been developed over the years including “fast track programs, and baccalaureate programs for practical nurses (LPN/RPN)(Canadian Association of Schools of Nursing, 2022), and are generally designed to provide a broad, foundational knowledge base, rather than in-depth knowledge of a focused area (Canadian Association of Schools of Nursing, 2022). Irrespective of which path the student chooses, the main focus is providing a program that is grounded in excellence in nursing education (Canadian Association of Schools of Nursing, 2022).

Nurses also have an ethical responsibility to maintain and utilize their knowledge and competence after graduation (Canadian Nurses Association, 2017). For practicing nurses, continuing nursing education (CNE) courses related to the work environment and factors associated with NSPD could be delivered through ongoing professional development (Bullock et al., 2020), with their health care organizations potentially providing funding and work release. To support their nursing staff, nursing managers might focus more on topics such as organizational culture, leadership, and conflict resolution to help foster and support an empowering work environment.

Finally, workplace restructuring is a common cause of job dissatisfaction and poor workplace morale (Klein & Hopper, 2013; Lim, 2014). Educating managers in change management methodologies might be helpful in mitigating morale issues arising from the often necessary and inevitably disruptive restructuring that is occurring in many health care systems (Turato et al., 2022).

## Conclusions and Next Steps

This two-city two-region study has provided a comprehensive analysis of work environments which might be used by health care managers to enhance NSPD. The evidence provided in this study can be used by health administrators and policy experts to devise improved strategies for creating optimal work environments for nurses working in Canadian hospitals.

There is a need to further understand factors associated with NSPD and to validate these findings through the use of multi-site studies of RNs and LPNs from all areas of care, not only cardiology and stroke. It is imperative to include studies that investigate linkages between patient outcomes and NSPD. Subsequent research could include other factors such as physical design of the nursing unit and the collective bargaining climate of the province in which the hospital is located.

Further research is needed to improve NSPD without increasing monetary perks. Health care budgets are constrained and collective bargaining agreements for nurses are becoming more rigid, reducing the discretion of health administrators to selectively reward nurses based on performance. Studies of satisfaction with performance of duties for other health professionals working in hospital settings might provide additional evidence on how best to support nurses and other health care workers in their efforts to improve patient outcomes.

## Notes

1. Also referred to as Registered Practical Nurses in the province of Ontario, Canada.

2. Healthcare facility recognized for excellence in nursing, quality patient care, and innovations in nursing practice by the American Nurses Credentialing Center.

3. A type of leadership based on integrity, honesty, high moral values and transparency when interacting with others to build trust and create an environment in which staff are engaged and motivated.

4. Refers to efforts to provide front line nurses with greater access to the information, resources, and opportunities for professional development needed to improve decision making and increase job performance.
